# Influence of computed tomography–based pelvimetric parameters and surgical approaches on surgical difficulty in mid‑low rectal cancer

**DOI:** 10.20452/wiitm.2025.

**Published:** 2025-07-04

**Authors:** Jie Wang, Dengyang Fang, Ruiqi Li, Yifan Cheng, Shuai Zhao, Jiajie Zhou, Zhen Tian, Chenkai Zhang, Yayan Fu, Yong Wang, Jun Ren, Daorong Wang

**Affiliations:** Northern Jiangsu People’s Hospital Affiliated with Yangzhou University, Yangzhou University, Yangzhou, Jiangsu, China; Northern Jiangsu People’s Hospital, Clinical Teaching Hospital of Medical School, Nanjing University, Yangzhou, Jiangsu, China; Northern Jiangsu People’s Hospital, Yangzhou, Jiangsu, China; Yangzhou Key Laboratory of Basic and Clinical Transformation of Digestive and Metabolic Diseases, Yangzhou, Jiangsu, China; General Surgery Institute of Yangzhou, Yangzhou University, Yangzhou, Jiangsu, China

**Keywords:** laparoscopic, mid‑low rectal cancer, pelvimetric parameters, robotic, surgical difficulty

## Abstract

**INTRODUCTION:**

Despite a lack of a well-defined concept of ‘pelvic difficulties’, pelvimetric parameters significantly influence surgical difficulty and outcomes in mid-low rectal cancer (MLRC).

**AIM:**

The objective of this study was to explore the influence of pelvimetric parameters and surgical approaches on the difficulty of surgical procedures in MLRC.

**MATERIALS AND METHODS:**

A retrospective analysis was performed at the Northern Jiangsu People’s Hospital, including patients with a diagnosis of MLRC who underwent total mesorectal excision between January 2016 and June 2023. We analyzed the pelvimetric parameters and perioperative data.

**RESULTS:**

The study cohort comprised a total of 1138 individuals. Based on the surgical difficulty score, 374 patients were assigned to the difficult surgery (DS) group, and 764, to the non-difficult surgery group. Patients in the DS group were stratified into 2 groups based on the surgical approach: the robot-assisted laparoscopic surgery (RLS) group with 78 patients, and the conventional laparoscopic surgery group, including 296 patients. Multivariable analysis results showed that age, sex, pelvic inlet anteroposterior diameters (PIAPD), pubic symphysis height, pelvic depth, and angle A were independent influencing factors for DS.

**CONCLUSIONS:**

Age, sex, PIAPD, pubic symphysis height, pelvic depth, and angle A were independent factors influencing DS in MLRC. In the DS group patients, RLS had certain advantages.

## INTRODUCTION

Colorectal cancer, a prevalent malignant neoplasm within the digestive system, holds the third position in terms of occurrence, and is the second leading cause of death among all types of cancers globally.^1^ Surgery is the primary treatment for colorectal cancer. As minimally-invasive technology continues to advance, conventional laparoscopic surgery (CLS) and robot-assisted laparoscopic surgery (RLS) have been increasingly used in the management of rectal cancer.^2^^,^^3^^,^^4^ Surgical resection principles for mid-low rectal cancer (MLRC) follow the total mesorectal excision (TME) procedure, which is difficult to perform, especially in patients with a “difficult pelvis.”^5^^,^^6^^,^^7^

The concept of a difficult pelvis was first proposed by obstetricians.^8^ Over the past few years, a growing number of colorectal surgeons have discovered that MLRC patients with a difficult pelvis experience longer operation time, more intraoperative blood loss, and even lower rectal mesenteric specimen integrity.^9^^,^^10^^,^^11^ However, the concept of a difficult pelvis remains poorly defined.

To date, very few studies have focused on pelvimetric parameters that influence surgical difficulty in MLRC. Existing research contains divergent opinions regarding this aspect.^10^^,^^11^^,^^12^ Assessment of surgical difficulty in MLRC through preoperative clinical parameters and development of individualized treatment plans can benefit patients.

**FIGURE 1:**
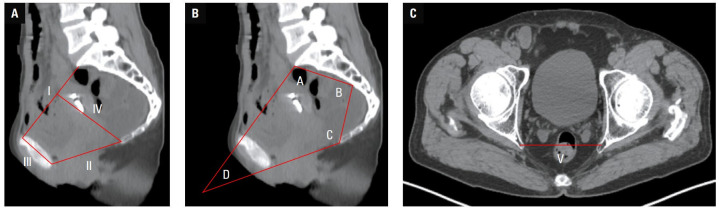
Computed tomography (CT)-­based pelvimetric parameters; A, B – pelvic sagittal CT images; pelvic inlet anteroposterior diameter (I); pelvic outlet anteroposterior diameter (II); pubic symphysis height (III); pelvic depth (IV); letters A–D represent pelvic angles. C – pelvic cross‑sectional CT image; bispinous diameter (V)

## AIM

The aim of this study was to investigate the impact of pelvimetric parameters and surgical approaches on the difficulty of surgical procedures in MLRC. Despite the absence of a clearly defined concept of ‘pelvic difficulties’, pelvimetric parameters are known to play a crucial role in determining the complexity and outcomes of surgical interventions in MLRC. Through a retrospective analysis of patients who underwent TME at the Northern Jiangsu People’s Hospital between January 2016 and June 2023, this study sought to identify key pelvimetric factors that contributed to surgical difficulty. Additionally, the study aimed to compare the outcomes of 2 different surgical approaches, RLS and CLS, in patients whose surgeries were categorized as difficult. The ultimate goal was to provide insights that could help surgeons better anticipate and manage challenges associated with MLRC surgery, thereby improve patient outcomes.

## MATERIALS AND METHODS

### Patient population

We collected retrospective data of individuals with MLRC who underwent TME between January 2016 and June 2023 at the Northern Jiangsu People’s Hospital in Jiangsu, China.

**FIGURE 2:**
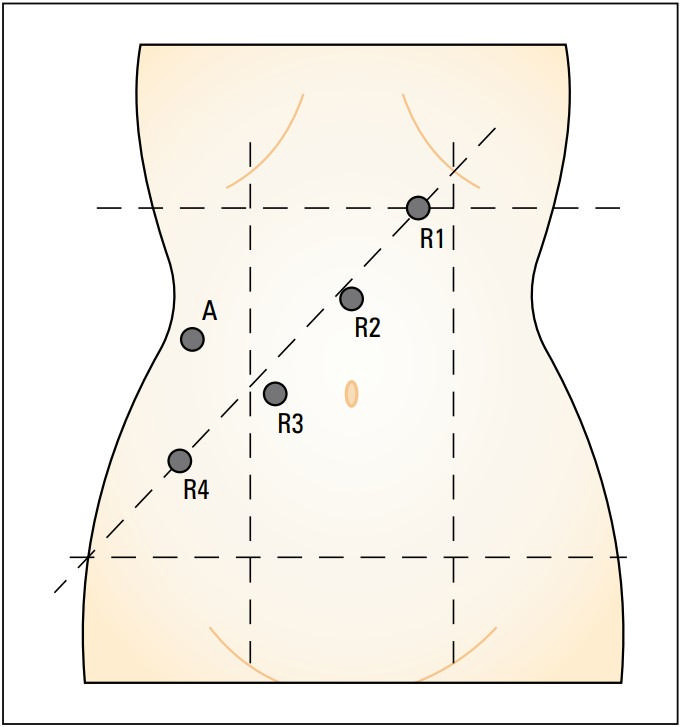
Location of robot‑assisted laparoscopic surgery ports; assistant port (A, 12 mm); robotic arms (R1, R2, R3, R4, 8 mm) with R2 used as a camera placed above the umbilicus

To be included in the study, the participants had to have a solitary malignant growth within the rectum, located within 10 cm of the anal verge, detected on endoscopy and computed tomography, and confirmed as malignant on pathological analysis. They also had to have undergone RLS or CLS.

Exclusion criteria comprised: emergency surgery due to acute abdominal symptoms, past diagnosis of malignancy, multiple primary tumors, history of abdominoperineal resection or Hartmann resection, and open surgery.

### Data collection

We retrospectively collected patients’ baseline and perioperative data, including: sex, age, prior abdominal surgery, American Society of Anesthesiologists (ASA) classification, body mass index (BMI), neoadjuvant therapy, surgical approaches, estimated blood loss, operation time, number of positive lymph nodes (LNs), number of harvested LNs, postoperative hospitalization, tumor distance from the anal verge, conversion to open surgery, overall postoperative complications, pathological tumor, node, metastasis (TNM) stage, perineural invasion, vascular invasion, and tumor differentiation. We classified total complications into grades I–II and III–IV based on the Clavien–Dindo system.^13^ Pelvimetric parameters were obtained from the sagittal and transverse CT sections (FIGURE 1), including pelvic inlet anteroposterior diameter (PIAPD), pelvic outlet anteroposterior diameter, pubic symphysis height, pelvic depth, bispinous diameter, AB line, BC line, angle A, angle B, angle C, and angle D.^14^^,^^15^^,^^16^ The difficult surgery (DS) group and the nondifficult surgery (NDS) group were established according to intraoperative and postoperative parameters, based on the study by Chen et al.^16^The comprehensive scoring was as follows: operation time below 170 minutes (1 point), conversion to open surgery (3 points), estimated blood loss below 100 ml (1 point), postoperative occurrence of Clavien–Dindo grade III–IV complications (2 points), and postoperative hospitalization below 15 days (1 point). The patients with total scores ranging from 0 to 2 were assigned to the NDS group, while the individuals who scored 3 or higher were placed in the DS group.

**FIGURE 3:**
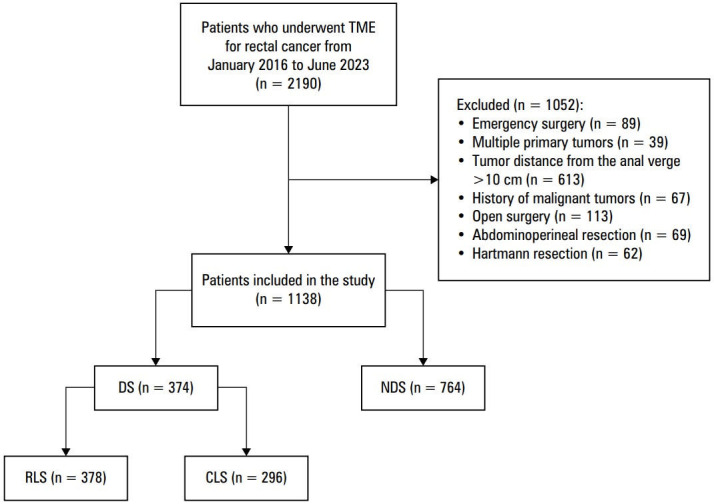
Clinical data selection flow chart Abbreviations: CLS, conventional laparoscopic surgery; DS, difficult surgery; NDS, nondifficult surgery; RLS, robot‑assisted laparoscopic surgery; TME, total mesorectal excision

**TABLE 1:** Basic characteristics of the patients

Parameter		DS (n = 374)	NDS (n = 764)	P value
Age, y		62.3 (7.8)	61.2 (8.8)	0.02
Sex, n (%)	Men	229 (61.2)	398 (52.1)	0.004
	Women	145 (38.8)	366 (47.9)	
BMI, kg/m²		22.3 (3.2)	22.6 (3.3)	0.07
Prior abdominal surgery, n (%)		41 (11)	88 (11.5)	0.78
ASA classification, n	I	152 (40.6)	281 (36.8)	0.43
	II	163 (43.6)	349 (45.7)	
	III	59 (15.8)	134 (17.5)	
Neoadjuvant therapy, n (%)		82 (21.9)	152 (19.9)	0.43
PIAPD, cm		11.2 (0.7)	11.3 (0.7)	0.02
POAPD, cm		8.6 (0.7)	8.7 (0.6)	0.83
Pubic symphysis, cm		4.8 (0.7)	4.7 (0.7)	2
Pelvic depth, cm		10.6 (0.9)	10.5 (0.8)	0.02
Bispinous diameter, cm		9.5 (0.8)	9.6 (0.7)	5
AB line, cm		7.7 (0.4)	7.8 (0.4)	0.66
BC line, cm		6.4 (0.5)	6.3 (0.6)	0.79
Angle A, °		88.2 (5.8)	89.5 (5.7)	<0.001
Angle B, °		117.9 (6.7)	118.2 (6.6)	0.54
Angle C, °		106.1 (6.2)	106.7 (6.3)	0.12
Angle D, °		46.6 (5.4)	46.5 (5.5)	0.8

Data are presented as mean (SD) unless indicated otherwise.

Abbreviations: BMI, body mass index; ASA, American Society of Anesthesiologists; PIAPD, pelvic inlet anteroposterior diameters; POAPD, pelvic outlet anteroposterior diameters; others: see FIGURE 3

### Surgical technique

The patients were administered general anesthesia and positioned in the modified lithotomy position with legs apart. The principle of TME was strictly adhered to intraoperatively, and LNs of group 253 were routinely cleared. The positioning of the ports for the Da Vinci Surgical Xi System (Intuitive Surgical, Sunnyvale, California, United Staes) is illustrated in FIGURE 2. The decision to preserve the left colic artery was made intraoperatively depending on the situation. After dissecting to the preresection plane, the rectal wall was exposed using an ultrasonic scalpel. Once the dissection reached the bottom of the tumor, the rectum was transected 2–3 cm away from the distal end of the tumor. After removing the tumor through the abdominal incision, the rectum was transected approximately 10 cm proximal to the upper edge of the tumor. The 2 ends of the intestine were anastomosed within the abdominal cavity using a circular stapler.

The laparoscopic procedure was similar to the robotic surgery. The camera port was positioned 3 cm superior to the umbilical midline. Based on the tumor’s location, the primary surgical trocar, equipped with a 12-mm port, was placed in the lower right quadrant. Another port was fixed 3 cm to the right of the umbilicus.

### Ethics approval statement

The study complied with the ethical standards set forth in the Declaration of Helsinki and was granted approval by the Ethics Committee of the Northern Jiangsu People’s Hospital (2019KY-22). All participants voluntarily agreed to take part in the study and signed the informed consent form.

### Statistical analysis

Statistical analysis was conducted using IBM SPSS Statistics package version 26.0 (IBM Corp., Armonk, New York, United States). Continuous data were depicted as mean (SD) or median (interquartile range [IQR]), and differences were assessed using the independent samples *t* test. Categorical data were presented as proportions and analyzed using the χ^2^ test. Logistic regression models were employed for univariable and multivariable analyses. Clinical parameters with a *P* value below 0.05 in the univariable analysis were selected for inclusion in the multivariable analysis. A *P* value below 0.05 was deemed significant.

**TABLE 2:** Intraoperative and postoperative outcomes

Parameter		DS (n = 374)	NDS (n = 764)	P value
Surgical approach	RLS	78 (20.9)	161 (21.1)	0.99
	CLS	296 (79.1)	603 (78.9)	
Operation time, min, mean (SD)		221.4 (21.5)	178.6 (25.2)	<0.001
Estimated blood loss, ml, mean (SD)		135.7 (24.6)	66.3 (20.6)	<0.001
Postoperative hospitalization, d, mean (SD)		16.6 (5.6)	9.0 (2.6)	<0.001
Number of harvested LNs		16 (14–22)	18 (14–24)	0.46
Number of positive LNs		1 (0–3)	1 (0–3)	0.15
Tumor distance from the anal verge, cm, mean (SD)		7 (1)	6.9 (1.1)	0.2
Overall postoperative complications		63 (16.8)	92 (12)	0.03
Clavien–Dindo grade I–II	Overall	34 (9.1)	66 (8.6)	0.89
	Wound infection	16 (4.3)	32 (4.2)	
	Urinary retention	11 (2.9)	21 (2.7)	
	Ileus	8 (2.1)	18 (2.4)	
Clavien–Dindo grade III–IV	Overall	31 (8.3)	28 (4.2)	2
	Anastomotic leakage	27 (7.2)	22 (3.7)	
	Postoperative bleeding	5 (1.3)	2 (0.3)	
Pathological T stage	T1	50 (13.4)	119 (15.6)	0.39
	T2	92 (24.6)	167 (21.9)	
	T3	165 (44.1)	319 (41.8)	
	T4	67 (17.9)	159 (20.7)	
Pathological N stage	N–	224 (59.9)	482 (63.1)	0.33
	N+	150 (40.1)	282 (36.)	
Pathological M stage	M–	363 (97.1)	732 (95.8)	0.39
	M+	11 (2.9)	32 (4.2)	
Perineural invasion		72 (19.3)	123 (16.1)	0.21
Vascular invasion		61 (16.3)	109 (14.3)	0.41

Data are presented as numbers and percentages or median (interquartile range) unless indicated otherwise.

Abbreviations: LN, lymph node; others, see FIGURE 3

## RESULTS

### Patient characteristics

Initially, 2190 individuals with rectal cancer were identified, but after excluding 1052 of them, the study ultimately included 1138 patients (FIGURE 3). Among these, the DS group had 374 patients, and the NDS group, 764 participants. The differences in age, sex, PIAPD, pubic symphysis height, pelvic depth, bispinous diameter, and angle A were significant between the 2 groups (*P* <⁠0.05). Other baseline characteristics were insignificant, as presented in TABLE 1.

### Perioperative outcomes

Mean (SD) operation time in the DS group was 221.4 (21.5) minutes, which was considerably longer than in the NDS group (178.6 [25.2] min; *P* <⁠0.001). The DS group experienced mean (SD) estimated blood loss of 135.7 (24.6) ml, which was notably greater than the amount observed in the NDS group (66.3 [20.6] ml; *P* <⁠0.001). Additionally, the DS group had markedly longer mean (SD) postoperative hospitalization in comparison with the NDS group (16.6 [5.6] vs 9 [2.6] d; (*P *<⁠0.001).

The overall postoperative complication rate for the DS group was 16.8%, as compared with 12% observed in the NDS group (*P* <⁠0.05). No noteworthy intergroup differences were observed for the remaining perioperative indicators (including surgical approach, number of harvested LNs, number of positive LNs, tumor distance from the anal verge, pathological TNM stage, perineural invasion, and vascular invasion; TABLE 2).

### Univariable and multivariable analysis of difficult surgery

The following clinical parameters were identified as potential factors contributing to DS in MLRC: age, sex, PIAPD, pubic symphysis height, pelvic depth, bispinous diameter, and angle A. TABLE 3 outlines the results obtained from both univariable and multivariable logistic regression analyses pertaining to DS. Age (odds ratio [OR], 0.984; 95% CI, 0.369–0.998; *P* = 0.03), sex (OR, 1.452; 95% CI, 1.129–1.868; *P* = 0.004), PIAPD (OR, 1.231; 95% CI, 1.028–1.475; *P* = 0.02), pubic symphysis height (OR, 0.75; 95% CI, 0.624–0.901; *P* = 0.002), pelvic depth (OR, 0.828; 95% CI, 0.707–0.969; *P* = 0.02), bispinous diameter (OR, 1.258; 95% CI, 1.071–1.478; *P* = 0.04), and angle A (OR, 1.039; 95% CI, 1.017–1.061; *P* = 0.001) were all associated with DS in MLRC. In the subsequent stepwise multivariable logistic regression analysis, age, sex, PIAPD, pubic symphysis height, pelvic depth, and angle A were identified as independent factors influencing DS in patients with MLRC.

### Stratified analysis of different surgical approaches in patients undergoing difficult surgery

#### Basic difficult surgery characteristics with different surgical approaches

The patients with MLRC in the DS group were further categorized into the RLS group (78 individuals) and the CLS group (296 individuals) based on the surgical approach. An intergroup comparison showed no significant differences regarding age, sex, BMI, prior abdominal surgery, ASA classification, tumor distance from the anal verge, tumor differentiation, and neoadjuvant therapy (TABLE 4).

**TABLE 3:** Univariable and multivariable analysis of difficult surgery

Parameter		Univariable analysis		Multivariable analysis	
		OR (95% CI)	P value	OR (95% CI)	P value
Age		0.984 (0.369–0.998)	0.03	0.981 (0.966–0.996)	0.01
Sex	Women	Reference	—	—	—
	Men	1.452 (1.129–1.868)	4	1.433 (1.108–1.854)	0.006
BMI		1.037 (0.598–1.079)	0.07	—	—
Prior abdominal surgery	No	Reference	—	—	—
	Yes	0.946 (0.638–1.401)	0.78	—	—
Neoadjuvant therapy	No	Reference	—	—	—
	Yes	1.131 (0.835–1.53)	0.43	—	—
Surgical approach	RLS	Reference	—	—	—
	CLS	1.013 (0.748–1.373)	0.93	—	—
PIAPD		1.231 (1.028–1.475)	0.02	1.254 (1.041–1.509)	0.02
POAPD		0.978 (0.804–1.191)	0.83	—	—
Pubic symphysis		0.75 (0.624–0.901)	2	0.748 (0.619–0.903)	0.003
Pelvic depth		0.828 (0.707–0.969)	0.02	0.846 (0.721–0.994)	0.04
Bispinous diameter		1.258 (1.071–1.478)	0.04	1.236 (1.048–1.458)	0.06
AB line		1.067 (0.801–1.422)	0.66	—	—
BC line		1.035 (0.806–1.328)	0.79	—	—
Angle A		1.039 (1.017–1.061)	1	1.041 (1.018–1.064)	<0.001
Angle B		1.006 (0.987–1.025)	0.54	—	—
Angle C		1.016 (0.996–1.036)	0.12	—	—
Angle D		0.997 (0.974–1.02)	0.79	—	—

Abbreviations: OR, odds ratio; others, see FIGURE 3 and TABLE 1

#### Perioperative outcomes in difficult surgery with different surgical approaches 

In the RLS group, mean (SD) estimated blood loss was considerably smaller than in the CLS group (128.5 [19.5] vs 162.7 [22.7] ml; P <0.001). Mean (SD) postoperative hospitalization was shorter in the RLS group, as compared with the CLS group (14.1 [4.4] vs 17.2 [5.7] d; P <0.001). No notable differences were observed between the 2 groups in terms of other perioperative clinical indicators (TABLE 5).

## DISCUSSION

Surgical treatment of MLRC remains a significant challenge in colorectal oncology, particularly due to the anatomical constraints imposed by the pelvic cavity. Although advancements in minimally-invasive techniques, such as laparoscopic and robot-assisted surgery, have improved outcomes, the inherent complexity of pelvic anatomy continues to impact surgical difficulty and postoperative results. This study systematically evaluated 11 pelvic parameters and identified 4 bony factors (PIAPAD, pubic symphysis height, pelvic depth, and angle A) as independent predictors of surgical difficulty in MLRC. Our findings underscore the critical interplay between pelvic morphology and technical feasibility, providing actionable insights for preoperative risk stratification and surgical planning.

Existing literature has recognized the association between pelvic anatomy and surgical difficulty in rectal cancer.^17^^,^^18^^,^^19^^,^^20^ However, the lack of consensus on quantitative definitions of a ‘difficult pelvis’ hampers clinical standardization. Early studies by Escal et al^17^ and Nagtegaal et al^19^ categorized surgical difficulty through composite end points, such as operative time, conversion rates, and intraoperative blood loss. However, these frameworks were tailored to open surgery and do not apply to contemporary minimally-invasive approaches. A recent work by Chen et al^16^ applied these metrics to laparoscopic surgery but omitted critical anatomic variables. A notable gap in previous research lies in the overreliance on soft tissue parameters (eg, mesorectal thickness), while neglecting bony structures. For instance, Baek et al^18^ concluded that there was no significant correlation between pelvimetric measurements and surgical difficulty; however, their single-center cohort (n = 45) and subjective difficulty classification limited statistical power. In contrast, our multivariable analysis of 1138 patients, incorporating patient demographics and multiplanar CT-derived pelvic measurements, showed nuanced relationships between bony structures and surgical outcomes.

Consistent with historical observations,^21^ male patients demonstrated higher surgical difficulty scores, attributable to the sexual dimorphism in pelvic anatomy. The narrower male pelvis reduces working space, complicating instrument maneuverability and visualization. This dimorphism aligns with evolutionary biomechanical adaptations, but presents unique challenges in oncologic resections requiring precise mesorectal dissection. Advanced age emerged as an independent risk factor, likely reflecting cumulative comorbidities (eg, cardiopulmonary disease) and reduced tissue elasticity. Frailty indices and sarcopenia, although not explored here, may synergize with pelvic anatomy to amplify intraoperative instability and prolong recovery times.

**TABLE 4:** Basic patient characteristics in difficult surgery with different surgical approaches

Parameter		RLS (n = 78)	CLS (n = 296)	P value
Age, y, mean (SD)		62.5 (7.4)	62.3 (7.9)	0.86
Sex	Men	49 (62.8)	180 (60.8)	0.75
	Women	29 (37.2)	116 (39.2)	
BMI, kg/m², mean (SD)		22.2 (4.5)	22.3 (3.1)	0.76
Prior abdominal surgery		9 (11.5)	32 (10.8)	0.86
ASA classification	I	27 (34.6)	122 (41.2)	0.35
	II	35 (44.9)	131 (44.3)	
	III	16 (20.5)	43 (14.5)	
Tumor distance from the anal verge, cm, mean (SD)		6.9 (1.1)	7 (1)	0.61
Tumor differentiation	Good	9 (11.5)	43 (14.5)	0.87
	Moderate	32 (41)	109 (36.8)	
	Poor	8 (10.3)	32 (10.8)	
	Mucous	29 (37.2)	112 (37.9)	
Neoadjuvant therapy		15 (19.2)	67 (22.6)	0.62

Data are presented as numbers and percentages unless indicated otherwise.

Abbreviations: see FIGURE 3 and TABLE 1

**TABLE 5:** Intraoperative and postoperative outcomes in difficult surgery with different surgical approaches

Parameter		RLS (n = 78)	CLS (n = 296)	P value
Operation time, min, mean (SD)		219.3 (25.3)	213.6 (21.5)	0.32
Estimated blood loss, ml, mean (SD)		128.5 (19.5)	162.7 (22.7)	<0.001
Postoperative hospitalization, d, mean (SD)		14.1 (4.4)	17.2 (5.7)	<0.001
Number of harvested LNs, median (IQR)		16 (14–25)	16 (14–22)	0.79
Number of positive LNs, median (IQR)		1 (0–3)	1 (0–3)	0.69
Overall postoperative complications		15 (19.2)	48 (16.2)	0.64
Clavien–Dindo grade I–II	Overall	8 (10.3)	26 (8.8)	0.86
	Wound infection	4 (5.1)	15 (5.1)	
	Urinary retention	3 (3.8)	8 (2.7)	
	Ileus	1 (1.3)	4 (1.4)	
Clavien–Dindo grade III–IV	Overall	7 (9)	24 (8.1)	0.99
	Anastomotic leakage	6 (7.7)	21 (7.1)	
	Postoperative bleeding	1 (1.3)	4 (1.4)	
Pathological T stage	T1	11 (14.1)	39 (13.2)	0.37
	T2	18 (23.1)	74 (25)	
	T3	30 (38.5)	135 (45.6)	
	T4	19 (24.4)	48 (16.2)	
Pathological N stage	N−	49 (62.8)	175 (59.1)	0.64
	N+	29 (37.2)	121 (40.9)	
Pathological M stage	M−	77 (98.7)	286 (96.6)	0.55
	M+	1 (1.3)	10 (3.4)	
Perineural invasion		14 (17.9)	58 (19.6)	0.87
Vascular invasion		11 (14.1)	50 (16.9)	0.67

Data are presented as numbers and percentages unless indicated otherwise.

Abbreviations: see FIGURE 3 and TABLE 1 and TABLE 2

Currently, there is a lack of consensus in the existing literature regarding the impact of pelvic measurements on surgical difficulty in rectal cancer procedures. Migaczewski et al^22^ included data from 110 patients undergoing rectal tumor surgery, and the results showed that pelvic measurement was not related to difficulties during laparoscopic surgery. Studies by Yamaoka et al^23^ and de’Angelis et al,^24^ on the other hand, showed that pubic symphysis height and pelvic depth correlated with difficult surgery in rectal cancer. Our study included 11 pelvimetric parameters, ultimately identifying 4 bony parameters as independent factors contributing to DS in MLRC. The synergistic or antagonistic effects between these 4 pelvimetric parameters may contribute to the development of a clinically challenging pelvic architecture. A longer pubic symphysis height shifts the surgical field deeper into the pelvis, requiring steeper laparoscopic angles and increasing instrument collision. Greater pelvic depth disproportionately affects the visualization of the presacral space, compromising distal tumor dissection. Narrow PIAPAD values and acute angle A reflect a compressed pelvic inlet, which restricts instrument triangulation—a cornerstone of minimally-invasive techniques. These parameters can dynamically synergize. For instance, a deep pelvis combined with a considerable symphysis height creates a “funnel effect”, exacerbating the spatial constraints of a narrow PIAPAD. These interactions may be nonlinear, warranting further geometric modeling.

With the increasing maturity of robotic technology, its application in rectal cancer has become more widespread. The advantages and disadvantages of RLS and CLS have been a topic of discussion among colorectal surgeons. Previous studies^2^**^,^**^7^**^,^**^25^ have shown that RLS has better tumor resection outcomes and a better short-term prognosis than CLS in rectal cancer. However, there is a scarcity of studies on RLS in the context of difficult surgeries in MLRC. This research evaluated and compared short-term outcomes of different surgical approaches in MLRC involving DS. The results showed lower estimated blood loss and shorter postoperative hospitalization in the RLS group than the CLS group. This outcome is consistent with the findings reported by Chang et al.^26^ Technological advantages, such as high field-of-view clarity, stability, and accuracy in precise manipulation may have positively influenced the RLS group. In terms of complications and the number of LNs harvested, the RLS group did not have a significant advantage over the CLS group. The findings match those observed in earlier studies.^27^**^,^**^28^ Clinical practice at our center has shown that both surgical approaches were viable DS options in MLRC, with robotic-assisted laparoscopic surgery offering certain advantages.

Our study advances prior work in 3 key areas. The first one is the multiparametric pelvic measurement. By integrating sagittal, coronal, and axial CT reconstructions, we captured the 3-dimensional (3-D) anatomical complexity more comprehensively than earlier studies reliant on 2-D measurements. We also contributed to the robust difficulty classification, showing that composite end points (conversion rates, blood loss, operative time, complications, hospital stay) balance surgical and patient-centered outcomes. Finally, our study integrated short-term RLS outcomes, which broadened the relevance of our findings to emerging technologies, where precise anatomical knowledge can mitigate learning curve effects. While our study provides novel anatomical insights, its limitations must be acknowledged. First, its retrospective single-center design introduces selection bias; prospective multi-institutional cohorts would enhance external validity. Second, the exclusion of pelvic soft tissue metrics (eg, visceral fat, mesorectal volume) may confound the impact of bony parameters. Future studies should integrate magnetic resonance imaging-based volumetric measurements and artificial intelligence-driven morphometric analyses to refine predictive models. Additionally, clinical trials comparing preoperative pelvic assessment protocols (eg, 3-D-printed models vs CT-guided planning) could optimize surgical preparation.

## CONCLUSIONS

Our study demonstrated that age, sex, PIAPD, pubic symphysis height, pelvic depth, and angle A were independent factors contributing to DS in MLRC. Longer pubic symphysis height, greater pelvic depth, shorter PIAPD, and narrower angle A may be useful in the preoperative assessment of a difficult pelvis in MLRC.

## JOURNAL INFORMATION

Videosurgery and Other Miniinvasive Techniques is an official journal of the Videosurgery Foundation.
